# Increased PD-1-positive macrophages in the tissue of gastric cancer are closely associated with poor prognosis in gastric cancer patients

**DOI:** 10.1186/s12885-020-6629-6

**Published:** 2020-03-04

**Authors:** Yusuke Kono, Hiroaki Saito, Wataru Miyauchi, Shota Shimizu, Yuki Murakami, Yuji Shishido, Kozo Miyatani, Tomoyuki Matsunaga, Yoji Fukumoto, Yuji Nakayama, Chiye Sakurai, Kiyotaka Hatsuzawa, Yoshiyuki Fujiwara

**Affiliations:** 1 Division of Surgical Oncology, Department of Surgery, School of Medicine, Tottori University Faculty of Medicine, 36-1 Nishi-cho, Yonago, 683-8504 Japan; 2 Department of Surgery, Japanese Red Cross Tottori Hospital, 117 Shotoku-cho, Tottori, 680-8517 Japan; 3 Division of Radioisotope Science, Research, Initiative Center, Organization for Research Initiative and Promotion, Tottori University, 86 Nishi-cho, Yonago City, Tottori 683-8503 Japan; 4 Division of Molecular Biology, Department of Molecular and Cellular Biology, School of Life Science, Faculty of Medicine, Tottori University, 86 Nishi-cho, Yonago, Tottori, 683-8503 Japan

**Keywords:** Gastric cancer, Macrophage, PD-1, Prognosis, Tumor immunity

## Abstract

**Background:**

Programmed cell death 1 (PD-1) is one of the immune checkpoint molecules that negatively regulate the function of T cells. Although recent studies indicate that PD-1 is also expressed on other immune cells besides T cells, its role remains unclear. This study aims to evaluate PD-1 expression on macrophages and examine its effect on anti-tumor immunity in gastric cancer (GC) patients.

**Methods:**

The frequency of PD-1^+^ macrophages obtained from GC tissue was determined by multicolor flow cytometry (*n* = 15). Double immunohistochemistry staining of PD-1 and CD68 was also performed to evaluate the correlations among the frequency of PD-1^+^ macrophages, clinicopathological characteristics, and prognosis in GC patients (*n* = 102).

**Results:**

The frequency of PD-1^+^ macrophages was significantly higher in GC tissue than in non-tumor gastric tissue. The phagocytotic activity of PD-1^+^ macrophages was severely impaired compared with that of PD-1^−^ macrophages. The 5-year disease-specific survival rates in patients with PD-1^+^ macrophage^Low^ (the frequency of PD-1^+^ macrophages; < 0.85%) and those with PD-1^+^ macrophage^High^ (the frequency of PD-1^+^ macrophages; ≥ 0.85%) were 85.9 and 65.8%, respectively (*P* = 0.008). Finally, multivariate analysis showed the frequency of PD-1^+^ macrophage to be an independent prognostic factor.

**Conclusions:**

The function of PD-1^+^ macrophage was severely impaired and increased frequency of PD-1^+^ macrophage worsened the prognosis of GC patients. PD-1–PD-L1 therapies may function through a direct effect on macrophages in GC.

## Background

The recent successes of immune checkpoint inhibitors in the treatment of various tumor types clearly indicate that immunotherapy is effective even in patients with cancer. The antibody against programmed cell death 1 (PD-1) is the most clinically successful immune checkpoint drug in the treatment for cancer patients [1–3]. Since PD-1 is closely associated with dysfunction of CD4^+^ and CD8^+^ T cells, the efficacy of the antibody against PD-1 is widely thought to be attributed to activation of T- cell in the treatment of cancer. However, the detailed mechanisms by which the anti-PD-1 antibody activates immunity against cancer cells have remained unclear.

Macrophages are immune cells belong to the innate immune system. They phagocytose bacteria and other harmful organisms and initiate inflammation by releasing pro- inflammatory mediators. They also present antigens to T cells and play important roles in cell-mediated immunity. A previous study reported that macrophages express PD-1 during pathogen infection [4–7]. Furthermore, Gordon et al. recently demonstrated that the function of tumor-associated macrophages (TAMs) that express PD-1 was impaired, which resulted in the progression of tumors [8], indicating that PD-1 was involved in the function of macrophages.

Gastric cancer (GC) is the third cause of cancer death worldwide [9]. We previously reported upregulated PD-1 expression on both CD4^+^ and CD8^+^ T cells obtained from cancer tissue in GC patients [10]. The function of these PD-1-positive CD4^+^ and CD8^+^ T cells was impaired, suggesting that increased frequency of PD-1^+^ T cells might play important roles in immune evasion of GC patients. Nivolumab, one of anti-PD-1 antibodies, was recently reported to be effective in the treatment of GC [11]. Given the fact that PD-1 expression is upregulated on both CD4^+^ and CD8^+^ T cells, the primary mechanism of the anti-PD-1 antibody in GC patients may be in the regulation of T cells. However, other effects of the anti-PD-1 antibody remain unclear thus far. It is indispensable to unveil the detailed mechanisms by which anti-PD-1 antibody activate anti-tumor immunity in cancer patients to maximize its effects and develop more effective cancer immunotherapy. Therefore, the current study was undertaken to evaluate PD-1 expression on macrophages in GC tissue and examine its effect on anti-tumor immunity in GC patients.

## Methods

### Patients

This study included gastric adenocarcinoma patients who underwent gastrectomy at Tottori University Hospital (Yonago, Japan). The patients who had preoperative treatment, such as radiotherapy, chemotherapy, or other medical interventions, were excluded. Adjuvant S-1 was performed in 34 patients who had stage II or III GC. The Japanese Classification of Gastric Cancer was used to determine the clinicopathologic findings [12]. This study was approved by the Institutional Review Board at Tottori University Hospital (18A108).

### Isolation of tumor-infiltrating mononuclear cells

Tumor-infiltrating mononuclear cells were isolated from 15 GC patients who underwent gastrectomy as previously described [13]. In brief, fresh cancer tissues and non-cancerous gastric mucosa (at least 5 cm apart from the tumor in the resected specimen) were cut into small pieces with a size of approximately 1 mm, and digested with 0.002% DNase I, 0.08% collagenase IV, and 0.01% hyaluronidase (all from Worthington, Lakewood, NJ, USA) at 37 °C for 60 min. After filtering through 70-μm cell strainers (BD Falcon, Franklin Lakes, USA), density-gradient centrifugation using Ficoll-Paque (Pharmacia, Uppsala, Sweden) was performed to obtain the mononuclear cells.

### Flow cytometry analysis

The antibodies used in this study are follows: anti-PD-1-phycoerythrin (PE) (Biolegend, San Diego, USA), anti-PD-1-peridinin-chlorophyll-protein complex (PerCP) (Biolegend), anti-CD45-PE-Cyanin 5 (PE-Cy5) (BD PharMingen, San Jose, USA), anti-CD11b-fluorescein isothiocyanate (FITC) (BD PharMingen), anti-CD11b-Allophecocyanin (APC) (BD PharMingen), anti-CD11c-APC (BD PharMingen), and anti-CD206-APC (BD PharMingen). The BD LSRFortessa™ cell analyzer (BD Biosciences, San Jose, CA, USA) was used for the analysis.

### Phagocytosis assay

CD11b-positive cells were isolated from mononuclear cells obtained from GC tissue using a Magnetic Cell Sorting System (Milteny Biotec, Bergisch Gladbach, Germany). Cells were resuspended into RPMI 1640 (Thermo Fisher Scientific, Tokyo, Japan) in 96 well plate (Corning, NY, USA) and incubated at 37 °C with Texas red conjugated Zymosan A (FUJIFILM, Tokyo, Japan) for 4 h. After washing with phosphate buffered salts (PBS; FUJIFILM), cells were stained with anti-CD11b-FITC, anti-PD-1-PerCP, and DAPI (Cell Biolabs, San Diego, CA, USA). The numbers of PD-1^+^ macrophages that phagocytosed Zymosan A were determined by flow cytometry analysis.

### Immunohistochemistry assay

Immunohistochemistry was carried out using samples from 102 patients with stage I–III gastric adenocarcinoma as previously described [13]. Four μm-thick paraffin sections were dewaxed, deparaffinized in xylene, and rehydrated through a graded alcohol series. The sections were boiled for 20 min in a microwave oven in 10 mM citrate buffer (pH 6.0) to retrieve PD-1 and CD68 antigen. The slides were subsequently incubated with rabbit anti-PD-1 antibody (Clone EPR4877(2), Abcam plc, Cambridge, UK; 1:500 dilution) and mouse anti-CD68 antibody (Clone PG-M1, Dako, Santa Clara, CA, USA; 1:100 dilution) overnight at 4 °C. The slides were then incubated with the conjugated goat anti-mouse polymer horseradish peroxidase (HRP) and the conjugated goat anti-rabbit polymer alkaline phosphatase (AP) secondary antibodies. (MACH 2 double stain®; Biocare Medical, Pacheco, CA, USA) for 30 min. Staining was visualized with peroxidase substrate (ImmPACT® DAB; Vector Laboratories, Burlingame, CA) and AP substrate (ImmPACT® Vector® Red; Vector Laboratories), which were visible as brown and red, respectively. The counterstain was then performed using Mayer’s hematoxylin solution (FUJIFILM). Images of 3 fields (× 200), which were randomly selected in a blinded manner, were acquired using a Nikon Eclipse Ts2 microscope (Nikon Instech, Tokyo, Japan). The separation of stains was achieved using the color deconvolution plug in of ImageJ software 1.47 (National Institutes of Health, USA) [14]. Using the cell counter plug in of ImageJ software, the number of stained cells was determined for each image. The frequency of PD-1^+^ macrophages was represented by the ratio of the number of PD-1^+^ CD68^+^ cells to that of CD68^+^ cells.

### Immunofluorescence staining

Immunofluorescence staining for PD-1 and CD68 was performed as previously described [15]. Four μm thick paraffin-embedded sections were incubated with primary antibodies, which were the same antibodies used in immunohistochemistry, overnight at 4 °C. The slides were then incubated with Goat Anti-mouse IgG H&L (Alexa Fluor® 488) and Goat Anti-rabbit IgG H&L (Alexa Fluor® 647) (Abcam plc., Cambridge, UK; 1:500 dilution) for 30 min at room temperature. After washing with PBS, slides were mounted with ProLong Gold antifade reagent with 4,6-diamidino-2-phenylindole (Thermo Fisher Scientific) and examined using a Nikon Eclipse Ts2 microscope (Nikon Instech).

### Statistical analysis

The differences between the frequency of PD-1^+^ macrophages in GC tissue and that in non-cancerous gastric tissue were compared by paired t-test. The differences of clinicopathologic characteristics between two groups were compared by Mann-Whitney U test. Receiver operating characteristic (ROC) analysis was used to determine the Youden index. The frequency of PD-1^+^ macrophages with the Youden index was used as an optimal cutoff value. Survival rates were calculated using the Kaplan-Meier method and their differences were determined using the log-rank test. Cox’s proportional hazards model was used to perform univariate analyses. Cox’s proportional hazards model and a stepwise procedure were used for multivariate analyses. A value of *P* < 0.05 was considered statistically significant. SPSS statistics version 24 (SPSS Inc., Chicago, IL, USA) and GraphPad Prism version 6 (GraphPad Software, Inc., La Jolla, CA, USA) software were used for all statistical analyses.

## Results

### PD-1 ^+^ macrophages are abundant and functionally impaired in GC tissue

**Fig. 1:**
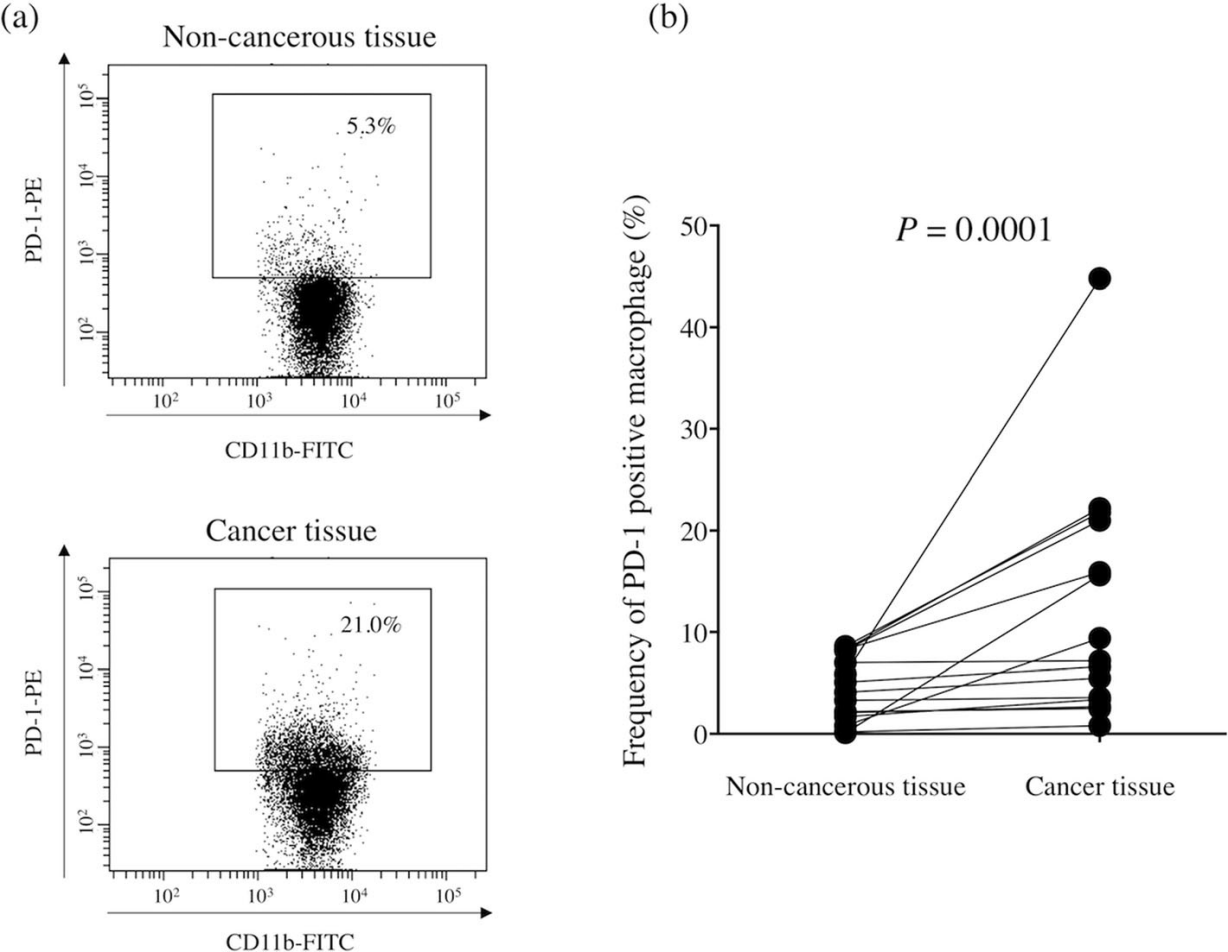
Presence of PD-1^+^ macrophages in gastric cancer tissue by flow cytometry. **a** Representative FACS data for PD-1 expression on macrophages obtained from non-cancerous gastric mucosa and gastric cancer tissue. **b** PD-1 expression is significantly higher on macrophages obtained from gastric cancer tissue than on those obtained from non-cancerous gastric mucosa (*P* = 0.0001)

**Fig. 2:**
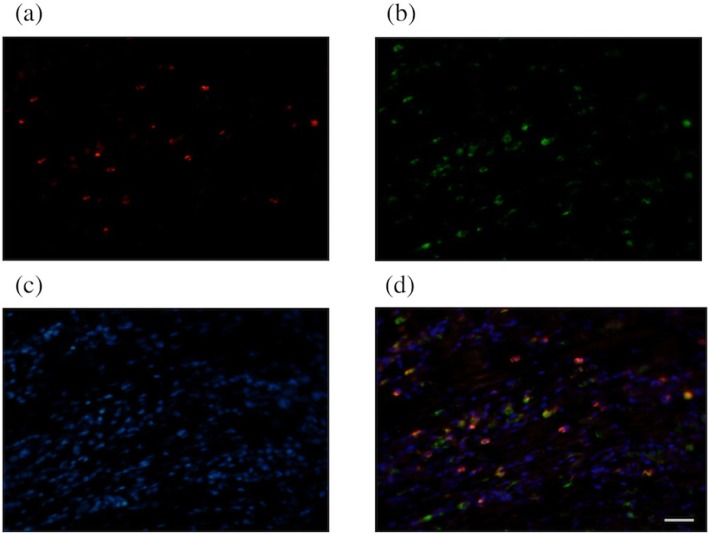
Presence of PD-1^+^ macrophages in gastric cancer tissue by immunofluorescence staining. Representative images of immunofluorescence staining of gastric cancer tissue for **a** PD-1, **b** CD68, **c** DAPI, and **d** merged staining

**Fig. 3:**
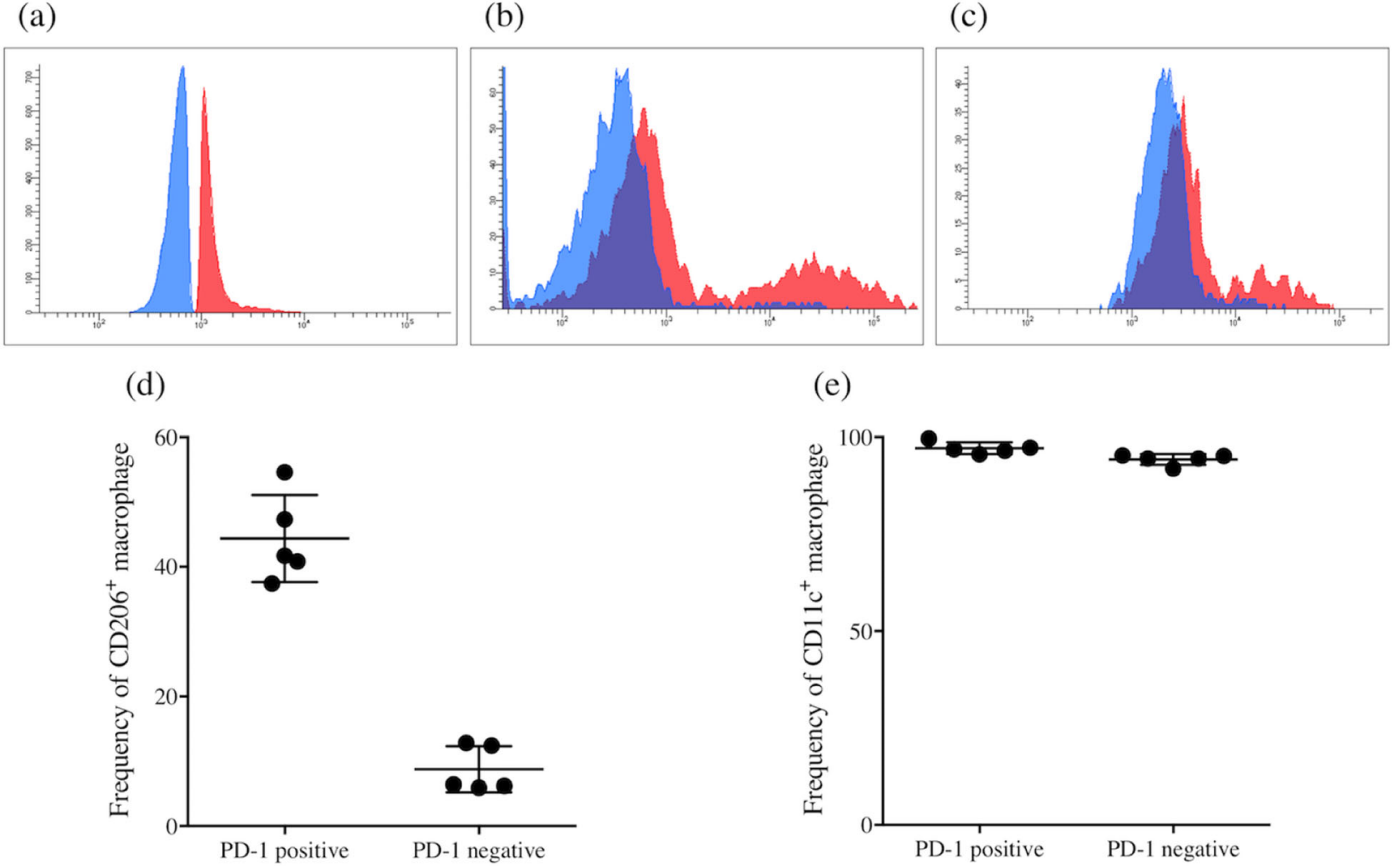
PD-1^+^ macrophages showed a trend towards the expression of an M2-like profile. Representative flow cytometry histograms showing expression of typical tumor-associated macrophage markers **a** PD-1; **b** CD206; **c** CD11c in PD-1^−^ versus PD-1^+^ macrophages in gastric cancer tissue (*n* = 5). Representative histograms are shown. Analysis of TAM markers **d** CD206; **e** CD11c in PD-1^−^ versus PD-1^+^ subsets from GC tissue shows that PD-1^+^ macrophages express more CD206 . *n* = 5, experiment conducted once. Paired one-tailed *t*-test

**Fig. 4:**
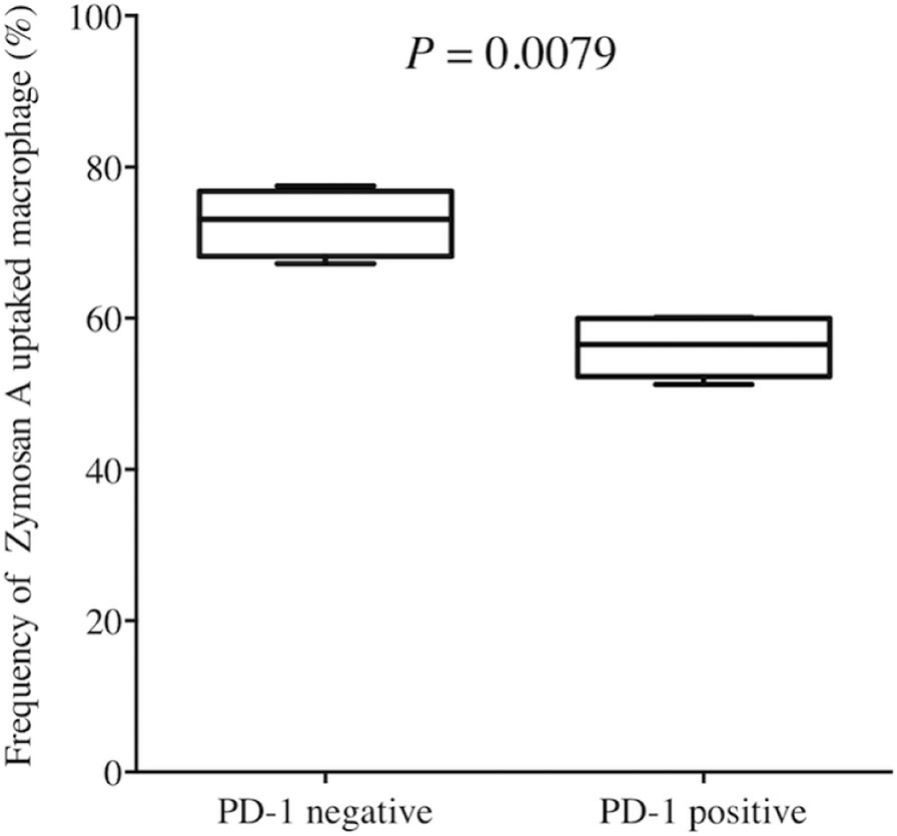
Comparison of the phagocytotic ability of PD-1^+^ macrophages and PD-1^−^ macrophages to Zymosan A. Zymosan A uptake by PD-1^+^ macrophage was significantly less than that by PD-1^−^ macrophages, indicating that the phagocytotic ability of PD-1^+^ macrophages obtained from GC tissue was impaired (*n* = 5)

We first determined the frequency of PD-1^+^ macrophages in GC tissue and non-cancerous gastric tissue by flow cytometry (*n* = 15). The frequency of PD-1^+^ macrophages was significantly higher in GC tissue than in non-cancerous gastric tissue (*P* = 0.0001, Fig. 1). Immunofluorescence staining also confirmed the presence of PD-1^+^ macrophages in GC tissue (Fig. 2). Flow cytometry analysis revealed that PD-1^+^ macrophages in GC tissue express more CD206, indicating that they showed an M2-like profile (Fig. 3). Therefore, PD-1^+^ macrophages in GC tissue seem to be pro-tumorigenic. Since a previous study demonstrated that the phagocytotic ability of PD-1^+^ macrophages was impaired in colorectal cancer [8], we next determined the phagocytotic ability of both PD-1^+^ and PD-1^−^ macrophages obtained from GC tissue using Zymosan A. Our results demonstrated that Zymosan A uptake by PD-1^+^ macrophages was significantly less than that by PD-1^−^ macrophages, indicating that the phagocytotic ability of PD-1^+^ macrophages obtained from GC tissue was impaired (*P* = 0.0079, Fig. 4).

### Increased number of PD-1^+^ macrophages is related to poor prognosis in GC patients

**Fig. 5:**
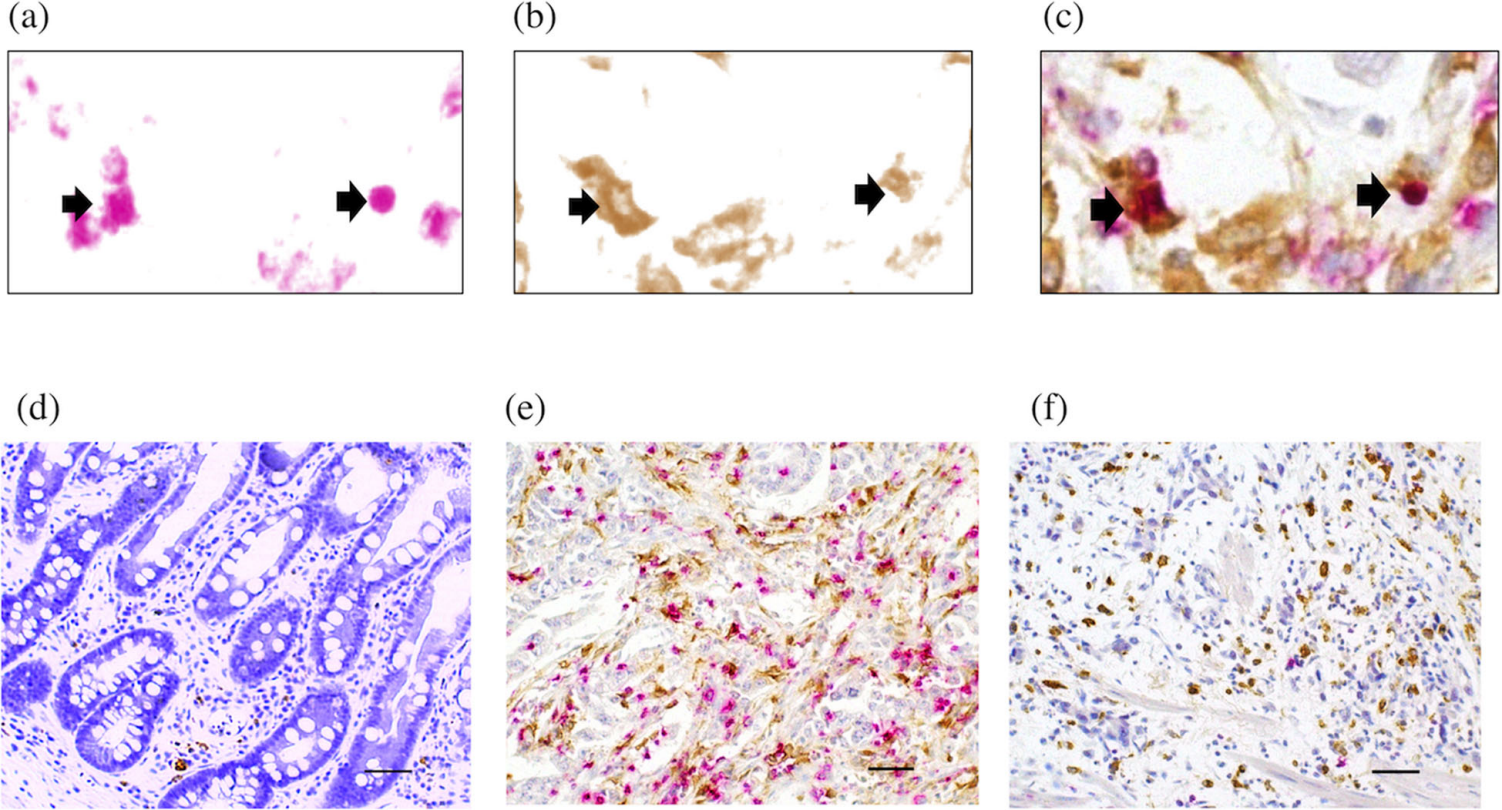
Presence of PD-1^+^ macrophages in normal gastric mucosa and gastric cancer tissue by immunohistochemistry. Representative image of PD-1^+^ macrophages (arrows) in gastric cancer tissue following double staining immunohistochemistry (pink, PD-1; brown, CD68). **a** PD-1, **b** CD68, **c** merge of PD-1 and CD68, **d** normal gastric mucosa, **e** gastric cancer tissue - PD-1^+^ macrophage^High^, (f) gastric cancer tissue - PD-1^+^ macrophage^Low^. Magnification 200×

The immunohistochemistry results revealed that there were PD-1 and CD68 double positive cells (PD-1^+^ macrophages) and PD-1^+^CD68^−^ cells, which were likely to be tumor-infiltrating lymphocytes, in GC tissues (Fig. 5). The frequencies of PD-1^+^ macrophages in GC tissue and in non-cancerous gastric tissue were 2.04 ± 2.77 and 0.0547 ± 0.131, respectively (*P* = 0.0001), which was consistent with the results by flow cytometry analysis. We then determined the correlations among the percentage of PD-1^+^ macrophages, clinicopathological variables and prognosis in GC patients (*n* = 102). The frequency of PD-1^+^ macrophages was significantly higher in patients aged 75 and more and those with lymph node metastasis than in patients aged less than 75 (*P* = 0.036) and those without lymph node metastasis (*P* < 0.001), respectively (Table 1).

**Table 1:** Relationships between the percentage of PD-1^+^ CD68^+^ cell and clinicopathological variables

	PD-1^+^ macrophage (%)	*P* value
Gender		0.24
Male (*n* = 75)	2.40 (±4.15)	
Female (*n* = 27)	1.24 (±2.45)	
Age		0.036
≥ 75 (*n* = 36)	3.31 (±4.93)	
< 75 (*n* = 66)	1.42 (±2.83)	
Depth of invasion ^a^		0.86
T1 (*n* = 12)	3.04 (±5.18)	
T2–4 (*n* = 90)	1.96 (±3.60)	
Lymph node metastasis		< 0.001
Absent (*n* = 54)	0.54 (±1.19)	
Present (*n* = 48)	3.83(±4.86)	
Tumor size		0.14
≥ 4 cm (*n* = 60)	2.48 (±3.97)	
< 4 cm (*n* = 42)	1.53 (±3.51)	
Histology ^b^		0.47
Differentiated (n = 54)	2.22 (±3.93)	
Undifferentiated (n = 48)	1.95 (±3.68)	
Lymphatic invasion		0.063
Absent (n = 12)	0.47 (±1.20)	
Present (n = 90)	2.30 (±3.97)	
Venous invasion		0.089
Absent (*n* = 17)	0.93 (±2.11)	
Present (*n* = 85)	2.32 (±4.02)	

All results expressed as mean ± SD

^a^Depth of invasion: T1: tumor has invaded lamina propria or submucosa; T2: tumor has invaded the muscularis propria; T3: tumor has invaded the subserosa; T4: tumor invasion is contiguous to, or exposed beyond, the serosa or has invaded adjacent structures

^b^Differentiated, papillary, or tubular adenocarcinoma; undifferentiated, poorly differentiated, mucinous adenocarcinoma, and signet-ring cell carcinoma

**Fig. 6:**
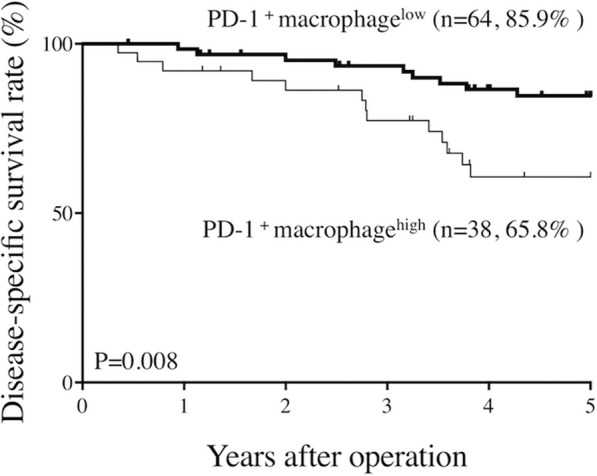
The prognosis of gastric cancer patients according to the frequency of PD-1^+^ macrophages. Five-year disease-specific survival rate of gastric cancer patients with a marked infiltration of PD-1^+^ macrophages was significantly lower than that of those with a slight infiltration of PD-1^+^ macrophages

ROC analysis indicated an optimal cutoff value with Youden index was 0.85%. Patients were then divided into PD-1^+^ macrophage (MAC) ^Low^ (< 0.85%) and PD-1^+^ MAC^High^ (≥ 0.85%) groups. Five-year disease-specific survival rates were also significantly higher in the PD-1^+^ MAC^Low^ group compared with the PD-1^+^ MAC^High^ group, at 85.9 and 65.8%, respectively (*P* = 0.008, Fig. 6). Univariate analysis revealed that lymph node metastasis, tumor size, and the frequency of PD-1^+^ macrophages were prognostic factors (Table 2). Finally, multivariate analysis revealed that the frequency of PD-1^+^ macrophages and tumor size were independent prognostic factors in GC patients (Table 2).

**Table 2:** Univariate and multivariate analyses of prognostic factors associated with disease-specific survival

Variables	Univariate analysis			Multivariate analysis		
	P value	HR	95% CI	P value	HR	95% CI
Age (≥75 vs. < 75)	0.53	1.293	0.578–2.895			
Gender (female vs. male)	0.29	0.578	0.222–1.557			
Tumor size (≥4 cm vs. < 4 cm)	0.004	4.111	1.553–10.879	0.010	5.001	1.470–17.008
Lymph node metastasis (present vs. absent)	0.010	2.864	1.284–6.388	0.21	1.843	0.701–4.841
Lymphatic invasion (present vs. absent)	0.17	4.117	0.558–30.378			
Venous invasion (present vs. absent)	0.063	6.679	0.905–49.289			
Depth of invasion (T1 vs. T2–4)	0.64	1.415	0.334–5.993			
Histology (undifferentiated vs. differentiated)	0.61	1.220	0.564–2.640			
PD-1+ CD68+ cell frequency (high vs. low)	0.008	2.971	1.369–8.131	0.031	2.560	1.087–6.026
S-1 adjuvant chemotherapy (absent vs. present)	0.022	2.671	*1.154–6.182*	*0.16*	1.546	0.581–4.116

See Table 1 for the detail of depth of invasion and histology

*CI* Confidence interval

*HR* Hazard ratio

## Discussion

We have demonstrated that certain portion of macrophages in GC tumor tissues express PD-1 in this study. The frequency of PD-1^+^ macrophages was more abundant in GC tissue than in non-cancerous gastric mucosa, suggesting the possibility that PD-1^+^ macrophages might play some important roles in the progression of GC. The frequency of PD-1^+^ macrophages by flow cytometry was more than that by immunohistochemistry in this study, possibly due to the different way of evaluation. PD-1 was first discovered as a molecule expressed on T cells that induced apoptosis of T cells [16] and then was identified as a co-signaling molecule by Honjo et al. [17]. PD-1 binds to either of its two ligands, PD-L1 or PD-L2, and delivers a co-inhibitory signal in T cells indicating that PD-1 negatively controls the function of T cells [17]. Although this molecule plays important roles in preventing hyperactivation of T cells, which is harmful for the host, it seems to be closely associated with immune evasion observed in chronic infections and tumors. In acute infections, PD-1 is upregulated upon T cell activation. After resolution of the infection, PD-1 expression on T cells decreases and T cells become memory T cells [18]. However, there are many exhausted viral-specific CD8^+^ T cells with high PD-1 expression in chronic human infections with HIV, HBV, and HCV. Although the function of these CD8^+^ T cells are severely impaired, recent work has shown that blockade of the PD-1 pathway can recover their function in vitro [19]. Furthermore, it was reported that tumor-infiltrating CD8^+^ T cells specific for tumor antigen, NY-ESO-1, increased PD-1 expression and their function was impaired in ovarian cancer patients [20]. In this regard, we previously reported that PD-1 expression on both CD4^+^ and CD8^+^ T cells obtained from cancer tissue was upregulated in GC patients and the function of these PD-1-positive CD4^+^ and CD8^+^ T cells was severely impaired. Furthermore, immunohistochemistry showed many PD-1^+^CD68^−^ tumor infiltrating cells, which were likely lymphocytes, in this study. These suggest that PD-1 expression on T cells is also related to immune evasion in cancer patients, including GC patients. Although most studies regarding immune evasion by PD-1 have focused on T cells, recent reports have demonstrated that other immune cells also express PD-1.

Natural killer (NK) cells paly important roles in the eradiation of cancer cells [21–23]. PD-1 overexpression was observed on peripheral and tumor-infiltrating NK cells from patients with digestive cancers including gastric cancer [24]. Blockade of the PD-1 pathway markedly enhances their cytokine production and suppresses their apoptosis, indicating that increased PD-1 expression was closely associated with dysfunction of NK cells. Furthermore, Xiao et al. recently identified a novel pro-tumorigenic B-cell subset with high PD-1 expression in human hepatocellular carcinoma. PD-1^hi^^gh^ B cells impaired the function of T-cell, which resulted in disease progression, via IL10-dependent pathways upon interacting with PD-L1 [25]. Overall, PD-1 overexpression on not only T cells but also other types of immune cells seems to be closely related to immune evasion observed in cancer patients.

Macrophages are typically divided into M1 and M2 phenotypes. M1-type macrophages kill target cells and produce inflammatory cytokines, indicating that they are anti-tumorigenic, whereas M2-type macrophages reduce inflammatory responses and adaptive Th1 immunity, indicating that they are pro-tumorigenic [26–29]. It has been demonstrated that TAMs polarize into the M2 phenotype and suppress the host immune responses against cancers, which results in tumor progression. Therefore, the presence of TAMs worsens prognosis in human cancers [30]. Our results revealed that PD-1^+^ macrophages showed an M2-like profile, indicating that PD-1^+^ macrophages are pro-tumorigenic. Our results further showed that the phagocytotic ability of PD-1^+^ macrophages was impaired compared with PD-1^−^ macrophages. The phagocytotic ability of macrophages plays an important role in preventing tumor progression. Therefore, it is likely that impaired phagocytotic ability of PD-1^+^ macrophages observed in the current study promotes tumor progression. In this regard, we also showed that the prognosis of GC patients with PD-1^+^ MAC^High^ was significantly worse than that of GC patients with PD-1^+^ MAC^Low^. Furthermore, multivariate analysis revealed that the frequency of PD-1^+^ macrophage was an independent prognostic indicator, indicating that the frequency of PD-1^+^ macrophage was closely associated with prognosis of gastric cancer patients regardless of stage of disease. Considering the close correlation between the frequency of PD-1^+^ macrophages and prognosis of GC patients, a therapeutic strategy targeting the phagocytotic activity of macrophages might be effective for the treatment of GC patients.

PD-1 binds to either PD-L1 or PD-L2, and delivers a co-inhibitory signal. We previously demonstrated that a certain proportion of GC cells expressed PD-L1 [31], indicating that PD-1 is able to deliver co-inhibitory signals in PD-1^+^ macrophages in GC patients.

Epstein-Barr virus (EBV) is an oncogenic human herpesvirus involved in the development of around 10% of GC. The overexpression of PD-L1 is one of the features of EBV-associated GC. Recent study demonstrated that EBV-associated gastric cancer cells expressing high levels of PD-L1 suppress T-cell proliferation [32]. Furthermore, PD-L1 expression on tumor-infiltrating immune cells was reported to be associated with distinct clinicopathological features, including high densities of tumor-infiltrating lymphocytes, mismatch repair deficiency, and EBV positivity in GC [33]. Helicobacter pylori infection, known as the strongest risk factor for GC, was also significantly associated with expression of PD-L1 and PD-1 [34]. However, the correlations among the frequency of PD-1^+^ macrophages, mismatch repair deficiency, and EBV and Helicobacter pylori infection remains unclear in this study. Therefore, further investigations are urgently required to unveil them.

## Conclusions

Our results suggest that PD-1 expression on macrophages is closely associated with their dysfunction in GC. Considering that upregulated PD-1 expression on macrophages is associated with poor prognosis, therapies targeting PD-1 pathway may function through a direct effect on not only T cells but also macrophages in GC.

## Data Availability

The datasets used and/or analysed during the current study are available from the corresponding author on reasonable request.

## Abbreviations

CD: Cluster of differentiation
CSF: Colony stimulating factor
DSS: Disease-specific survival
EBV: Epstein-Barr virus
FITC: Fluorescein isothiocyanate
GC: Gastric cancer
HBV: Hepatitis B virus
HCV: Hepatitis C virus
HIV: Human immunodeficiency virus
IL: Interleukin
MAC: Macrophage
NK: Natural killer
PBS: Phosphate buffered salts
PD-1: Programmed cell death 1
PerCP: Peridinin chlorophyll protein
RPMI: Roswell Park Memorial Institute
ROC: Receiver operating characteristic
TAMs: Tumor-associated macrophages
